# Revealing the Flavor Characteristics of Beiwudu Hulatang Using Electronic Nose, Electronic Tongue Combined with GC-IMS and Sensory Analysis

**DOI:** 10.3390/foods14234054

**Published:** 2025-11-26

**Authors:** Jing Yan, Heng Wang, Zhenxia Cao, Bing Yang, Wanli Zhang, Minnan Liu, Fazheng Ren, Lishui Chen

**Affiliations:** 1 Food Laboratory of Zhong Yuan, Luohe 462300, China; yanjing@zyfoodlab.cn (J.Y.); wangheng@zyfoodlab.cn (H.W.); c_zx0313@163.com (Z.C.); 18437902080@139.com (B.Y.); wlzhang916@163.com (W.Z.); liuminnan2021@163.com (M.L.); renfazheng@263.net (F.R.); 2 Key Laboratory of Precision Nutrition and Food Quality, Department of Nutrition and Health, China Agricultural University, Beijing 100193, China

**Keywords:** Beiwudu Hulatang, flavor profile, HS-GC-IMS, odor activity value (OAV), sensory correlation

## Abstract

Beiwudu Hulatang, a traditional Chinese culinary delicacy, is valued for its complex flavor profile; however, its characteristic aroma compounds and the determinants of sensory quality remain insufficiently studied. This study evaluated the flavor characteristics of four commercial samples and one laboratory-made sample of Beiwudu Hulatang using gas chromatography–ion mobility spectrometry (GC-IMS), electronic nose (E-nose), electronic tongue (E-tongue), and sensory evaluation. The results of E-tongue analysis indicated that bitterness and saltiness were the dominant taste attributes. E-nose analysis demonstrated strong responses to sulfur-containing compounds, alcohols, and alkanes, indicating their significant contribution to the overall aroma. A total of 60 volatile compounds were identified by GC-IMS, with ethers, alcohols, and terpenes being the most abundant chemical groups. Among these, 13 key aroma compounds were screened as discriminative markers (OAV > 1, VIP > 1) by integrating the odor activity value (OAV) and orthogonal partial least squares-discriminant analysis (OPLS-DA). The Pearson correlation analysis further revealed that sensory attributes, particularly aroma and overall acceptability, were positively correlated with propanal, heptaldehyde, and 1,8-cineol, and negatively correlated with linalool and limonene. Overall, this study provides a systematic characterization of the flavor profile of Beiwudu Hulatang and establishes a scientific basis for its quality standardization and flavor-oriented product development.

## 1. Introduction

Beiwudu Hulatang has long been one of the most distinctive traditional foods of Henan Province, China [1]. It is well-known for its complex preparation process that incorporates more than 20 medicinal and edible ingredients, including white pepper, black pepper, star anise, and Sichuan pepper, which produce a savory, moderately spicy broth with a lingering aroma. Traditionally, it is consumed for its purported health benefits such as improved digestion, spleen invigoration, and relief of cold-related symptoms [2]. Regional variations in Hulatang exist across Henan. The Beiwudu style, also known as “Stewed Meat Hulatang”, is characterized by tender meat, pronounced aromatic spiciness, and a rich aftertaste. As a characteristic local snack of Luohe City, it has a documented history dating back to the late Northern Song Dynasty [3]. Recently, Beiwudu Hulatang has gained increasing popularity for its distinctive flavor. Its sensory properties originate from multiple sources, for example, the abundant spice composition provides aroma and bioactive compounds, whereas the simmering of bone broth and meat releases low–molecular-weight flavor precursors [4]. In addition, thermal processing steps, such as the early stir-frying process and the later high-temperature treatment, induce flavor development [5,6], collectively forming the complex flavor profile of Hulatang.

The flavor profile of Beiwudu Hulatang is a critical determinant of its quality; however, systematic studies on investigations of its flavor compounds remain limited. Although previous research has demonstrated that HS-GC-IMS can effectively differentiate regional Hulatang varieties [7], systematic characterization of the flavor profile of Beiwudu Hulatang is still lacking. Although group standards for Beiwudu Hulatang have been well-established [8,9,10], providing certain benchmarks for quality control, its flavor evaluation continues to rely primarily on sensory assessment. Moreover, the key aroma-active compounds and their correlation with sensory attributes in Beiwudu Hulatang have not been elucidated yet. In addition, most existing studies have used single analytical methods, lacking the multi-method approach required for comprehensive flavor mapping, thereby limiting the depth of flavor profiling. Variations in raw materials and processing conditions may further contribute to inconsistencies in product quality, posing challenges to standardization and industrial-scale production. Flavor significantly influences sensory properties, with aroma and taste being the key factors affecting consumer preference [11]. Thus, identifying the characteristic flavor compounds is essential for stabilizing and enhancing product quality. Several multivariate statistical approaches such as principal component analysis (PCA), orthogonal partial least squares-discriminant analysis (OPLS-DA), and cluster analysis have been used to characterize the volatile compound profiles associated with specific sensory attributes [12].

So far, various techniques have been developed for flavor analysis, including gas chromatography (GC), gas chromatography–mass spectrometry (GC-MS), olfactometry, electronic nose (E-nose), electronic tongue (E-tongue), and gas chromatography–ion mobility spectrometry (GC-IMS) [13,14,15]. However, comprehensive characterization of food flavor remains challenging because of the complexity, structural diversity, and wide concentration range of volatile compounds—factors that exceeds the capabilities of any single analytical method [16,17]. Among these approaches, GC-IMS has emerged as a promising tool for separation and detection, offering high sensitivity and strong separation capability. It has been widely applied to detect volatile compounds, conduct quality control, and classify food products [18,19,20].

In this study, four commercially successful brands and a laboratory-made sample were selected based on market survey and consumer feedback to accurately characterize the flavor profile of Beiwudu Hulatang. Their flavor characteristics were analyzed using E-nose, E-tongue, and GC-IMS to establish a flavor fingerprint. The key volatile compounds were further identified by OPLS-DA and odor activity value (OAV) analysis, and their correlations with sensory attributes were investigated. This study provides a theoretical basis for evaluating the flavor profile of Beiwudu Hulatang.

## 2. Materials and Methods

### 2.1. Materials and Instruments

Beef, gluten block, beef tallow, sweet potato vermicelli, corn starch, and seasonings (including edible salt, monosodium glutamate, and spice powder) were purchased from a local supermarket. The specific brand information for the four commercial Beiwudu Hulatang samples is listed in Table 1. The C4-C9 2-ketones standard (2-butanone, 2-pentanone, 2-hexanone, 2-heptanone, 2-octanone, and 2-nonanone, all analytical grade) and 2-octanol (analytical grade) were supplied by Aladdin Industrial Corporation (Shanghai, China) and Shanghai Yuanye Bio-Technology Co., Ltd. (Shanghai, China), respectively. High-purity nitrogen gas (99.999%) was used. Headspace vials (20 mL) were procured from Hanon Instruments (Jinan, China). An MXT-WAX capillary column (15 m × 0.53 mm, 1.0 μm) was obtained from Restek Corporation (Pennsylvania, PA, USA).

The electronic tongue (SA402B) was obtained from Insent Co., Ltd. (Atsugi, Japan). The electronic nose (PEN3) was from AirSense Analytics GmbH (Schwerin, Germany). A FlavourSpec^®^ gas chromatography-ion mobility spectrometry (GC-IMS) system, equipped with a CTC-PAL 3 automatic headspace sampler (CTC Analytics AG, Zwingen, Switzerland) and an MXT-WAX capillary column (15 m × 0.53 mm, 1.0 μm; Restek, USA), was supplied by G.A.S. (Dortmund, Germany). Data acquisition was performed using the VOCal software (version 0.4.03, G.A.S., Germany).

### 2.2. Sample Preparation

Based on market research and consumer feedback, four types of Beiwudu Hulatang products were selected and labeled as D, W, G, and S; all samples were beef-flavored, individually packaged, and randomly chosen from batches with identical production dates.

The laboratory-made Beiwudu Hulatang (sample Z) was prepared according to the raw material and process specifications outlined in the group standard T/BWDHLT 001-2024 [8]. The process flow diagram is shown in Figure S1. Beef was boiled in water, skimmed, cooled, and diced into 1–2 cm pieces. Beef tallow was melted, followed by the sequential addition of diced beef, salt, monosodium glutamate, spice powder (including white pepper, star anise, Sichuan pepper, galangal, cardamom, amomum, clove, angelica, long pepper, tsao-ko, fennel, and dried tangerine peel), and gluten block. The mixture was simmered in a pot, cooled completely at 4 °C, portioned, vacuum-packaged into sauce packets, and sterilized at 121 °C for 20 min. The final product was assembled with sweet potato vermicelli and corn starch packets. All products were stored under ambient conditions. Prior to instrumental or sensory analysis, the samples were prepared according to the manufacturer’s instructions: water was added to a pot, followed by the sauce packet. After boiling, sweet potato vermicelli was incorporated, and the mixture was thickened with starch and cooked for 1–2 min. The resulting soup was then collected as the sample for subsequent analysis.

### 2.3. Electronic Tongue Analysis

The analysis was performed following the method of Yao et al. [21] with slight modifications. Briefly, 20 g of Hulatang sample was diluted with purified water at a 1:11 ratio, filtered, and heated to 50 °C in a water bath. Measurement was conducted using a SA402B taste sensing system equipped with sensors for sourness, bitterness, saltiness, umami, and astringency, along with two standard electrodes. After sensor activation and calibration, each sample was measured four times. The first measurement cycle was discarded, and the average of the last three cycles was used for data analysis.

### 2.4. Electronic Nose Analysis

A 10 g sample of Hulatang was placed in a headspace vial and incubated at 65 °C for 15 min. The measurement conditions were set as follows: sample interval 1 s, sensor cleaning time 80 s, sample injection time 5 s, injection flow rate 400 mL/min, and acquisition time 80 s. Each sample was analyzed in triplicate, with three consecutive measurements per replicate. Descriptions of the sensors used are provided in Table 2.

### 2.5. Analysis of Volatile Compounds by GC-IMS

For GC-IMS analysis, 2 g of Hulatang sample was accurately weighed into a 20 mL headspace vial, supplemented with 20 μL of 100 ppm 2-octanol solution as an internal standard, and incubated at 60 °C for 15 min with 500 rpm agitation prior to injection. Each sample was measured in triplicate under the following instrumental conditions: injection volume 500 μL in splitless mode with an injection needle temperature of 85 °C; the separation was performed on an MXT-WAX capillary column (15 m × 0.53 mm, 1.0 μm) held at 60 °C using high-purity nitrogen (≥99.999%) as carrier gas with a programmed flow rate starting at 2.0 mL/min for 2 min, ramped to 10.0 mL/min over 8 min, then to 100.0 mL/min in 10 min, and maintained for 10 min (total run time 30 min); the IMS detection utilized a tritium ionization source, a drift tube length of 98 mm, an electric field strength of 500 V/cm, a drift tube temperature of 45 °C, and high-purity nitrogen (≥99.999%) as drift gas at a flow rate of 150 mL/min under positive ion mode.

### 2.6. Odor Activity Value (OAV) Calculation

The odor activity value (*OAV*), defined as the ratio of the concentration of a volatile compound to its odor threshold in water, was used to evaluate the contribution of individual compounds to the overall aroma. *OAV* was calculated as follows:

$$OAV\;=\frac{Ci}{Ti}$$

*Ci* is the concentration of the volatile compound (μg/kg) and *Ti* is its odor threshold in water (μg/kg).

### 2.7. Sensory Evaluation

Thirty food science students (aged 20–30 years) without rhinitis or smoking habits were recruited and trained for sensory analysis. Participants were screened using triangle tests, in which they were required to identify the odd sample among three coded presentations (two identical, one different) [22]. A total of 20 panelists selected (10 male and 10 female). All assessments were conducted in a sensory evaluation laboratory under controlled conditions. Panelists were instructed on the evaluation objectives and procedures prior to analysis. Five freshly prepared Beiwudu Hulatang samples were presented in random order in white plastic cups, each labeled with a three-digit code. Panelists evaluated the samples based on appearance, aroma, texture, taste, and overall acceptability, using the criteria outlined in Table 3. Palates were cleansed with purified water between samples.

### 2.8. Statistical Analysis

HS-GC-IMS data were processed using the Laboratory Analytical Viewer (LAV) software (VOCal 0.4.10) with three dedicated plugins and the GC × IMS library (NIST and IMS databases), where the Reporter and Gallery Plot plugins were employed to generate three-dimensional and two-dimensional volatile compound spectra. Statistical significance was determined using The SAS 9.1.2 System for Windows V8, with data expressed as mean ± standard deviation and a *p*-value < 0.05 considered statistically significant. A semi-quantitative analysis of volatile compounds was performed by comparing peak areas with the internal standard (2-octanol) using the formula: Compound content (μg/kg) = (Peak area of compound × Amount of internal standard)/Peak area of internal standard. Multivariate statistical analysis, including OPLS-DA and variable importance in projection, was conducted using SIMCA 14.1, while Pearson correlation analysis was performed with IBM SPSS Statistics 26.0. Visualization was implemented using online and commercial tools: PCA and correlation plots were generated with https://www.chiplot.online/ (accessed on 2 July 2025), the venn diagram was created through the online website https://jvenn.toulouse.inrae.fr/app/example.html (accessed on 8 September 2025), radar charts were created using Origin 2018, and bar graphs were produced with GraphPad Prism 8.0.

## 3. Results and Analysis

### 3.1. Taste Profile Analysis of Beiwudu Hulatang by Electronic Tongue

**Figure 1:**
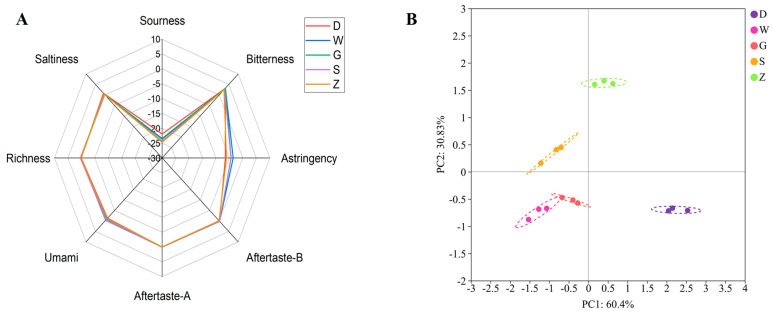
Electronic tongue radar charts (**A**) and PCA diagram (**B**) of different brands of Beiwudu Hulatang.

The taste characteristics of the 5 Beiwudu Hulatang samples were evaluated using an E-tongue, and radar plots (Figure 1A) were generated based on sensor responses, including sourness, bitterness, saltiness, astringency, bitterness aftertaste, astringency aftertaste, umami, and richness. A reference solution containing KCl and tartaric acid was used to define the taste baseline, with designated neutral points of −13 for sourness, −6 for saltiness, and 0 for other attributes [23]. Values above the neutral point indicate perceptible taste intensity. Overall, all samples exhibited positive responses to saltiness and bitterness, whereas sourness and astringency values showed negative responses. Umami, richness, and aftertaste attributes remained near neutral. These results suggest that saltiness and bitterness are the dominant taste attributes in Beiwudu Hulatang. The bitterness may originate from alkaloids and polyphenols present in spices such as pepper and Sichuan pepper [24]. Prolonged heating or imbalanced spice formulations can promote the oxidation of these compounds, enhancing bitter notes [25]. The laboratory-made sample exhibited lower bitterness and negative aftertaste values, likely because of the differences in ingredient selection and processing. Principal component analysis (PCA) of the E-tongue data revealed clear sample separation (Figure 1B). PC1 and PC2 accounted for 60.40% and 30.83% of the variance, respectively, cumulatively explaining 91.23% of the total variation. Samples W and G were clustered closely, indicating similar taste profiles, whereas the laboratory-made sample was clustered near the commercial sample S.

### 3.2. Flavor Profile Analysis of Beiwudu Hulatang by Electronic Nose

**Figure 2:**
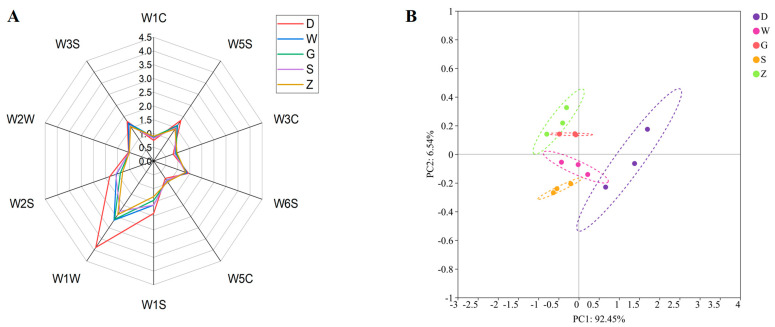
Electronic nose radar diagrams (**A**) and PCA diagram (**B**) of different brands of Beiwudu Hulatang.

The overall flavor profiles of the samples were characterized using E-nose [26]. As shown in Figure 2A, all five samples of Beiwudu Hulatang exhibited notable responses to sensors W5S (nitrogen oxides), W1S (methane), W1W (sulfides compounds and terpenes), W2S (alcohols and aromatic), and W3S (alkenes), indicating the potential significant contribution of these compounds to the distinctive aroma of Beiwudu Hulatang. Sample D showed markedly higher response values compared with other samples. The similar radar plot shapes among the samples suggest a shared flavor profile, although variations in the composition and concentration of specific volatile compounds were evident. The highest response was observed for sensor W1W (sulfides compounds and terpenes), whereas the lowest observed for W1C (aromatic compounds), indicating that the sulfur-containing and terpene compounds represent the dominant volatile group, whereas aromatic compounds appeared to contribute minimally. However, given the known cross-sensitivity of PEN3 sensors, this interpretation requires confirmation through chemical analysis. Subsequent GC-IMS analysis (Section 3.3) confirmed the presence of sulfur-containing and terpene compounds, thereby validating this preliminary finding. PCA of the E-nose data (Figure 2B) revealed clear separation among samples. PC1 and PC2 accounted for 92.46% and 6.54% of the variance, respectively, cumulatively explaining 99.00% of the total variation. Samples Z and G, along with W and S were closely positioned, with all samples distributed within the central region and showing overlapping confidence ellipses. This pattern reflects partial similarity in volatile composition among the five samples, suggesting a shared aroma foundation with subtle brand-specific variations.

### 3.3. Analysis of Volatile Compounds in Beiwudu Hulatang by HS-GC-IMS

**Figure 3:**
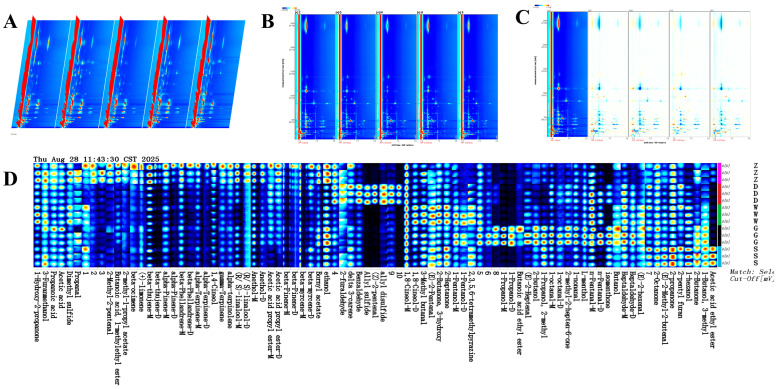
GC-IMS analysis of different brands of Beiwudu Hulatang. (**A**) 3D-topographic plots. (**B**) 2D-topographic plots. (**C**) Difference diagram. (**D**) Fingerprints of volatile compounds.

The volatile flavor compounds (VOCs) in the samples of Beiwudu Hulatang were characterized using HS-GC-IMS. The three-dimensional (3D) GC-IMS spectra (Figure 3A) display the ion migration time (*X*-axis), GC retention time (*Y*-axis), and signal intensity (*Z*-axis), clearly revealing the differences in VOCs among the samples. As shown in Figure 3A,B, the background of the spectra appears blue, and the red vertical line at migration time 1.0 corresponds to the reaction ion peak (RIP). Each dot on either side of the RIP represents a volatile compound. The signal intensity increases from blue to red, with darker colors indicating higher concentrations of the corresponding compound [27]. The laboratory-made sample (Group Z) was used as a reference to generate a differences map for clearer visualization of the differences in VOC among samples (Figure 3C). A white background indicates consistency with the reference, whereas red and blue represent higher or lower concentrations, respectively. The difference map for Sample D showed the closest VOC profile to that of group Z.

**Figure 4:**
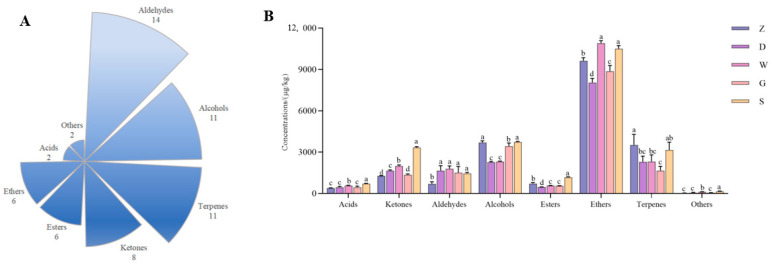
(**A**) Species diagram of the volatile compounds of Beiwudu Hulatang from different brands. (**B**) The content of the volatile compounds of Beiwudu Hulatang from different brands. Letters a–d indicate significant differences among the four groups (*p*  <  0.05).

A total of 86 signal peaks were detected across the 5 Beiwudu Hulatang samples (Table S1), of which 60 volatile compounds were identified (counting monomers and dimers once). These comprised 2 acids, 8 ketones, 14 aldehydes, 11 alcohols, 6 esters, 6 ethers, 11 terpenes, and 2 other compounds (Figure 4A). Aldehydes, alcohols, and terpenes were the most abundant chemical groups. Terpenes, primarily derived from spices such as pepper, Sichuan pepper, and star anise, contribute significantly to the aroma profile. Aldehydes, such as hexanal, octanal, pentanal, and heptanal, derived from the oxidation of unsaturated fatty acids [28], a process likely driven by the thermal processing of beef tallow and meat in Beiwudu Hulatang. Alcohols are mainly derived from the oxidative decomposition of lipids. Straight-chain monohydric alcohols impart green, fruity, and fatty-floral notes, whereas other characteristic aromas such as camphoraceous, minty, and spicy are more likely derived from the spices themselves [29]. Although only 6 ether compounds were identified (Figure 4A), they exhibited the highest relative content, followed by terpenes (Figure 4B). Ethers included sulfur-containing and oxygen-containing compounds. Sulfur ethers, typically exhibiting garlic, onion, or vegetable notes [30], likely originate from ingredients such as garlic and onion added during the initial stir-frying step. Most oxygen-containing ethers are volatile compounds extracted from spices [29]. Terpenes, characterized by low odor thresholds, also play a crucial role in the flavor formation of Beiwudu Hulatang [31]. In contrast, alcohols generally have high odor thresholds and contribute less to the overall flavor.

A volatile compound fingerprint was established from all identified compounds in the GC-IMS spectra (Figure 3D). Each row represents all signal peaks from 1 sample, whereas each column indicates the signal intensity of the same volatile compound across different samples. Notably, the laboratory-made sample Z contained higher concentrations of 2-methyl-2-pentenal, butanoic acid 1-methylethyl ester, 2-methyl-1-propyl acetate, β-ocimene, limonene, and β-thujene. Sample D had higher concentrations of 2-furaldehyde, benzaldehyde, allyl disulfide, (Z)-2-pentenal, and allyl sulfide. In sample W, 1,8-cineole and 3-methyl butanal were notably higher, whereas sample G was characterized by higher concentrations of 1-propanol, butanoic acid ethyl ester, and (E)-2-heptenal. Sample S exhibited prominent levels of 2-octanone, (E)-2-methyl-2-butenal, 2-propanone, 2-pentylfuran, 3-methyl-1-butanol, and acetic acid ethyl ester. These distinct profiles of characteristic compounds may serve as a basis for discriminating between different brands of Beiwudu Hulatang.

### 3.4. OAV Analysis of Aroma Compounds in Beiwudu Hulatang

**Figure 5:**
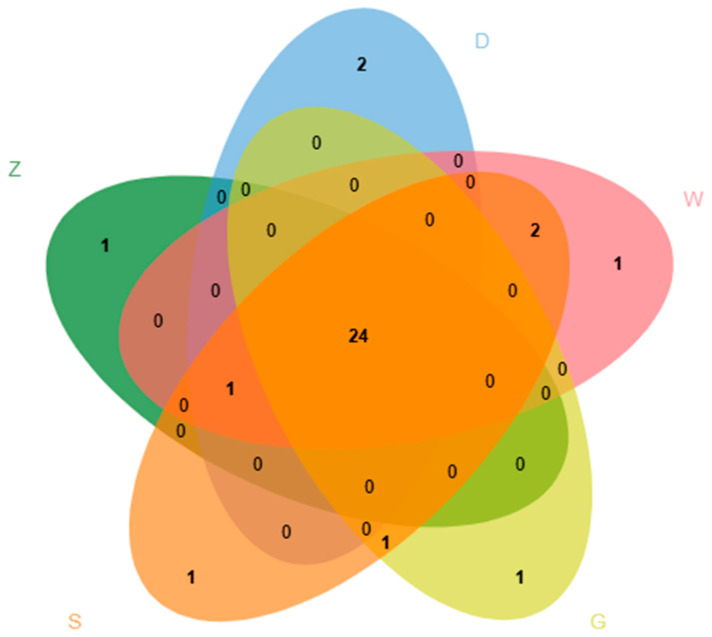
Venn diagram of volatile compounds with a ROAV ≥ 1 of Beiwudu Hulatang from different brands.

The OAV defined as the ratio of the concentration of a volatile compound to its odor threshold, is widely used to evaluate the contribution of individual compounds to the overall aroma profile [32]. Generally, an OAV ≥ 1 indicates a significant contribution to the overall aroma, with higher OAVs reflecting greater individual impact [33]. It should be noted that the OAVs reported here represent the estimated values derived from the semi-quantitative concentration data obtained using a single internal standard and published odor thresholds measured in water. These values require careful interpretation because of potential matrix effects in the complex Beiwudu Hulatang system and variations in detector response factors across different chemical classes. Our findings can provide a reference for future comparative studies with other traditional foods or complex matrices. A total of 34 key volatile compounds with estimated OAV > 1 were identified as potentially significant contributors (Table 4), of which 24 were common in all 5 samples (Figure 5). 1,8-Cineole was the predominant aroma contributor, imparting herbal, camphoraceous, and eucalyptus-like notes [34]. Other notable compounds with high estimated OAVs included anethole, β-myrcene, 1,4-cineole, β-phellandrene, and α-pinene, which collectively contributed to the spicy and herbal aromas [35,36,37]. Dimethyl sulfide contributed to vegetable-like notes [30], whereas compounds such as octanal, heptanal, nonanal, and 2-pentylfuran contributed to fatty, green, and citrus undertones [38,39]. Floral and fruity notes were attributed to linalool, acetic acid ethyl ester, 3-methyl-1-butanol, and limonene [36,40,41]. In addition, 3-methylbutanal contributed malty and almond-like characteristics [42], and pentanal contributed to fermented, yogurt, and pungent notes [38]. Several other compounds with estimated OAV > 1, including 3-hydroxy-2-butanone, (E)-2-heptenal, 1-hexanol, ethyl butanoate, bornyl acetate, β-pinene, and α-terpinene, also potentially served as important modifiers to the overall aroma profile of Beiwudu Hulatang.

### 3.5. Orthogonal Partial Least Squares-Discriminant Analysis (OPLS-DA) of Volatile Compounds

**Figure 6:**
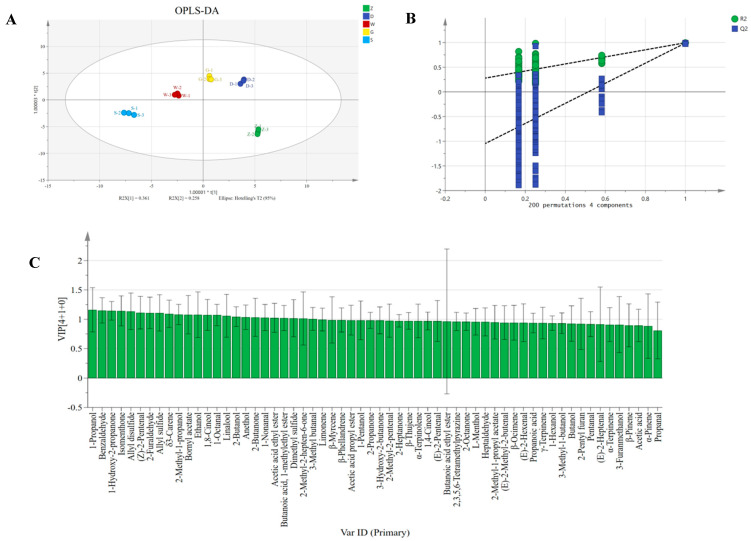
OPLS-DA analysis of Beiwudu Hulatang from different brands based on GC-IMS. (**A**) OPLS-DA score plot. (**B**) Model cross-validation results. (**C**) VIP scores of OPLS-DA.

Orthogonal partial least squares-discriminant analysis (OPLS-DA) was employed to extract meaningful information from the volatile compounds and to elucidate differences among the samples [43]. The interpretability (R^2^Y) and predictive capability (Q^2^Y) of the model, the latter derived through internal cross-validation, were evaluated using the R^2^X (0.961), R^2^Y (0.997), and Q^2^Y (0.987) values, respectively, demonstrating exceptional model fit and predictive performance (Figure 6A). A permutation test with 200 iterations further validated the model, with the Q^2^ regression line intercepting the vertical axis below 0 (−1.05), confirming no overfitting (Figure 6B). Variable importance in projection (VIP) values were used to assess the contribution of each volatile compound to the overall aroma profile. Compounds with VIP > 1.0 are generally considered discriminative markers [44]. As shown in Figure 6C, 24 volatile compounds were identified as significant contributors (VIP > 1, *p* < 0.05), including 5 alcohols, 1 terpene, 6 aldehydes, 4 ketones, 3 esters, and 5 ethers. The VIP scores were integrated with odor activity values (OAV > 1) to identify the following key aroma compounds in Beiwudu Hulatang: isomenthone, 2-methyl-2-hepten-6-one, 3-methyl butanal, 1-octanal, 1-nonanal, linalool, acetic acid ethyl ester, bornyl acetate, allyl disulfide, anethol, allyl sulfide, 1,8-cineol, and dimethyl sulfide.

### 3.6. Correlation Between Sensory Attributes and Characteristic Aroma Compounds

**Figure 7:**
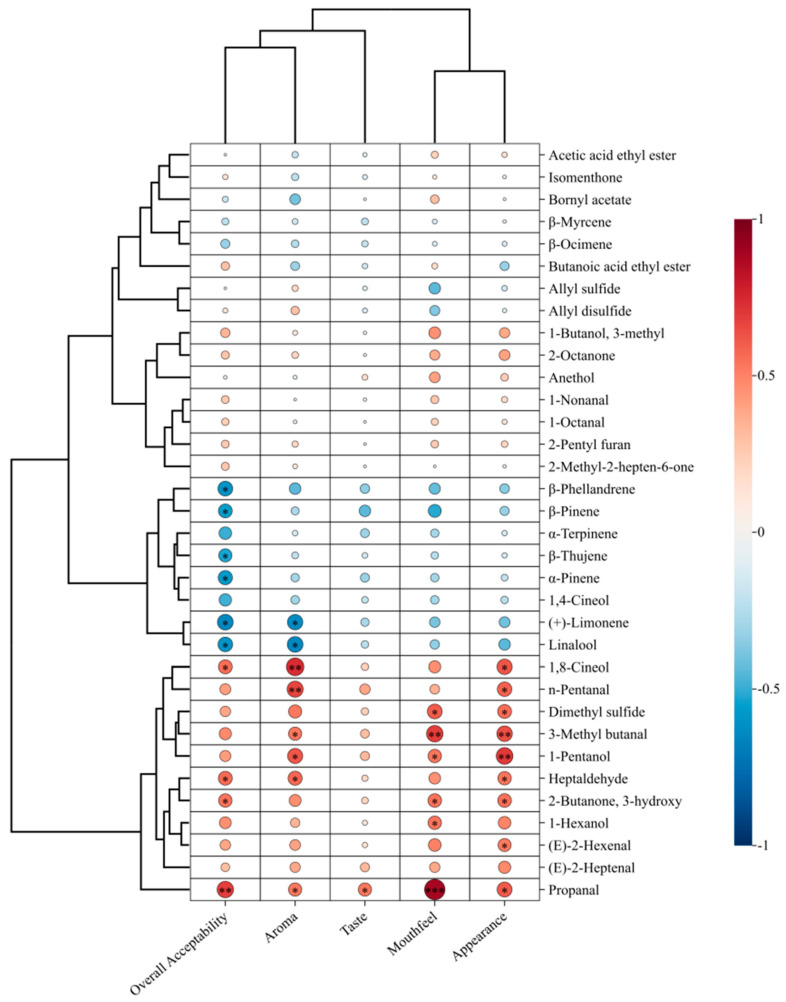
Pearson correlation map between the relative content of flavor substances in Beiwudu Hulatang and their and sensory evaluation. Note: Each circle represents the Pearson correlation coefficient (R), where * indicates *p* < 0.05, ** indicates *p* < 0.01, and *** indicates *p* < 0.001. Positive (0 < R < 1) and negative (−1 < R < 0) correlations are denoted by red and blue colors, respectively.

Based on the established methodologies that emphasize the complementary use of instrumental and sensory techniques [44,45], the sensory-related flavor compounds in Beiwudu Hulatang were identified through systematic sensory evaluation. Sensory evaluation revealed significant differences in 5 sensory attributes among the Beiwudu Hulatang samples (Table 5). The laboratory-made sample received significantly lower aroma scores compared with the commercial brands, whereas the samples from groups D and W were rated slightly higher in overall acceptability. The Pearson correlation analysis was performed to investigate the relationships between sensory scores (appearance, aroma, mouthfeel, taste, and overall acceptability) and the concentration of flavor compounds with OAV > 1 (Figure 7). The aroma scores showed significant positive correlations with propanal, heptaldehyde, 1-pentanol, 3-methylbutanal, pentanal, and 1,8-cineole (*p* < 0.05), and significant negative correlation with linalool and limonene (*p* < 0.05). Overall acceptability was positively correlated with propanal, 3-hydroxy-2-butanone, heptaldehyde, and 1,8-cineole, but negatively correlated with linalool, limonene, α-pinene, β-thujene, β-pinene, and β-phellandrene. As summarized in Table 4, the positively correlated compounds generally impart green, fatty, almond, and balsamic-fruity notes. In contrast, the most negatively correlated compounds were terpenes (e.g., linalool and limonene), which impart woody, citrus, mint, and camphoraceous characters and are likely derived from spices such as dried tangerine peel, clove, cinnamon, and fennel [36]. Notably, high concentrations of linalool may introduce a pungent odor [46], which could partially explain the lower aroma scores observed in certain samples. These correlation patterns provide valuable insights into practical applications in the food industry. For quality control purposes, the identified key aroma markers particularly the positively correlated compounds (propanal, heptaldehyde, and 3-hydroxy-2-butanone) can serve as quantitative indicators to monitor product consistency and authenticity. In product development, the correlation results offer scientific guidance for optimizing formulations; for instance, moderating the concentrations of negatively correlated terpenes (linalool, limonene) while enhancing the positively correlated aldehydes and 1,8-cineole could improve consumer acceptability. The similar correlation patterns observed between the key aroma compounds and both aroma and overall acceptability scores suggest that aroma characteristics not only define the sensory profile but also serve as critical determinants of consumer preference [47,48]. However, Pearson correlation analysis alone cannot establish causality between volatile compounds and sensory attributes. The practical application of these correlation findings requires further validation through sensory recombination studies (e.g., omission tests) or model system investigations.

## 4. Conclusions

Flavor characteristics are critical determinants of the quality of Beiwudu Hulatang. This study comprehensively analyzed the volatile composition and sensory attributes of 4 commercial brands and 1 laboratory-made sample using GC-IMS, E-nose, E-tongue, and sensory evaluation. The results showed significant correlations between sensory attributes and instrumental data. The results of E-tongue analysis indicated that bitterness and saltiness were the dominant taste attributes, followed by richness, bitter aftertaste, astringent aftertaste, and umami. The results of E-nose detection revealed strong responses to W5S (nitrogen oxides), W1S (methane), W1W (sulfides compounds and terpenes), W2S (alcohols and aromatics), and W3S (alkanes), with distinct aroma profiles among the samples. GC-IMS analysis detected 86 signal peaks and identified 60 volatile compounds, including 2 acids, 8 ketones, 14 aldehydes, 11 alcohols, 6 esters, 6 ethers, 11 terpenes, and 2 other compounds. A total of 34 key aroma compounds (OAV > 1) were screened by integrating OAV and OPLS-DA (VIP > 1), of which 24 compounds were common across all samples and 13 were identified as the key discriminators (OAV > 1 and VIP > 1). Pearson correlation analysis showed significant positive correlations between aroma and overall acceptability scores and the concentrations of propanal, heptaldehyde, and 1,8-cineole, whereas linalool and limonene were negatively associated with these sensory attributes. This study provides a comprehensive characterization of the flavor profile of Beiwudu Hulatang, offering valuable insights for product standardization and quality control. Study limitations include the relatively small sample size and the semi-quantitative nature of the GC-IMS data. Future work should focus on expanding sample sets, developing mechanistic models for flavor-formation pathways, and establishing quantitative relationships between processing parameters and flavor compound generation. These findings lay a foundation for quality standardization and targeted product optimization of Beiwudu Hulatang.

## Figures and Tables

**Table 1:** Sample information of four kinds of commercially available Beiwudu Hulatang.

Sample Number	Brand	Manufacturer	Origin
D	Beiduandu Dinguohua	Ding Guohua Hulatang Restaurant, Wuyang County	Luohe, China
W	Laowanjia	Lao Wan Jia Hulatang Restaurant, Wuyang County	Luohe, China
G	Shanguoyu	Shan Guoyu Hulatang Ingredient Processing Workshop, Wuyang County	Luohe, China
S	Shanshidingxingzhai	Shanshidingxingzhai Hulatang Restaurant, Wuyang County	Luohe, China

**Table 2:** Type and performance description of electronic nose system sensor.

Sensor Number	Sensor Name	Description of Main Applications
1	W1C	sensitive to aromatic compounds
2	W5S	sensitive to nitrogen oxides
3	W3C	sensitive to ammonia and aromatic compounds
4	W6S	mainly sensitive to hydrogen
5	W5C	sensitive to alkenes and aromatic compounds
6	W1S	sensitive to methane
7	W1W	sensitive to sulfides compounds and terpenes
8	W2S	sensitive to alcohols, partially aromatic compounds
9	W2W	sensitive to aromatic compounds and organic sulfides
10	W3S	mainly sensitive to alkenes

**Table 3:** Sensory evaluation criteria of Beiwudu Hulatang from different brands.

Indicator Evaluation	Scoring Criteria	Score Range
Appearance	Deep brown-yellow, uniform color, no stratification.	7~9
	Yellowish-brown, relatively uniform color, slight stratification.	4~6
	Light brown, non-uniform color, obvious stratification.	1~3
Aroma	Intense aroma, no off-odors, distinct characteristic aroma of Hulatang.	7~9
	Characteristic aroma of Hulatang is slightly faint, no off-odors.	4~6
	Lacks the characteristic aroma of Hulatang.	1~3
Mouthfeel	Smooth, fine, soft, non-irritating.	7~9
	Slightly coarse or gritty sensation; mildly irritating.	4~6
	Coarse and gritty; distinctly irritating.	1~3
Taste	Well-balanced spiciness and saltiness, rich and distinct flavor layers.	7~9
	Spiciness or saltiness slightly strong or weak, and moderate flavor layers.	4~6
	Overpowering spiciness or saltiness, lacks flavor layers.	1~3
Overall Acceptability	Like	7~9
	Neutral	4~6
	Dislike	1~3

**Table 4:** The contents and OAV of the volatile compounds in Beiwudu Hulatang from different brands.

Count	Compound Name	Formula	Aroma Attributes	Odor Threshold (μg/kg) ^A^	Concentration (μg/kg)					OAV				
					Z	D	W	G	S	Z	D	W	G	S
	Acids (2)													
1	Acetic acid	C2H4O2	Sour	22,000.00	340.60 ± 21.38	385.23 ± 50.19	484.78 ± 30.51	371.04 ± 75.7	602.57 ± 30.96	0.02	0.02	0.02	0.02	0.03
2	Propanoic acid	C3H6O2	Yogurt, vinegar	2190	58.20 ± 6.01	76.44 ± 6.43	87.34 ± 3.01	80.13 ± 10.43	115.13 ± 3.96	0.03	0.03	0.04	0.04	0.05
	Ketones (8)													
3	1-Hydroxy-2-propanone	C3H6O2	Pungent, caramel, fresh	10,000.00	97.13 ± 3.19	80.42 ± 3.95	143.41 ± 11.11	44.52 ± 2.73	109.37 ± 9.86	0.01	0.01	0.01	<0.01	0.01
4	2-Propanone	C3H6O	Pungent, irritating, floral	40,000.00	1028.90 ± 11.44	1209.79 ± 55.71	1525.72 ± 64.85	876.09 ± 49.72	2595.67 ± 53.19	0.03	0.03	0.04	0.02	0.06
5	2-Butanone	C4H8O	Acetone like, fruity, camphor	35,400.20	25.11 ± 3.16	15.81 ± 0.21	23.31 ± 0.83	37.72 ± 0.78	69.85 ± 6.28	<0.01	<0.01	<0.01	<0.01	<0.01
6	2-Butanone, 3-hydroxy	C4H8O2	Buttery, green, fatty	14.00	41.11 ± 4.76	47.41 ± 2.56	88.05 ± 5.51	43.26 ± 1.77	88.83 ± 10.95	2.94	3.39	6.29	3.09	6.35
7	Isomenthone	C10H18O	Mint, must, bitter	170.00	13.46 ± 1.57	161.61 ± 4.34	27.51 ± 0.77	213.56 ± 12.2	267.66 ± 8.77	0.08	0.95	0.16	1.26	1.57
8	2-Methyl-2-hepten-6-one	C8H14O	Citrus, fruity, moldy, ketone	68.00	25.35 ± 4.57	65.12 ± 2.23	50.48 ± 3.10	80.88 ± 0.99	44.99 ± 5.21	0.37	0.96	0.74	1.19	0.66
9	2-Octanone	C8H16O	Moldy, ketone, milk, cheese, mushroom	50.20	27.4 ± 1.09	31.41 ± 0.57	38.31 ± 0.84	28.61 ± 1.26	50.75 ± 1.92	0.55	0.63	0.76	0.57	1.01
10	2-Heptanone	C7H14O	Pear, banana, fruity, slight medicinal fragrance	140.00	27.79 ± 1.24	46.13 ± 3.05	94.52 ± 4.50	42.42 ± 0.60	104.21 ± 7.64	0.20	0.33	0.68	0.30	0.74
	Aldehydes (14)													
11	3-Methyl butanal	C5H10O	Malty, almond	1.10	102.70 ± 24.82	70.51 ± 10.94	218.03 ± 18.90	95.46 ± 33.93	197.01 ± 14.07	93.37	64.10	198.21	86.78	179.10
12	Pentanal	C5H10O	Fermented, yoghourt, pungent,	12.00	327.22 ± 105.62	771.96 ± 303.32	1013.66 ± 185.15	869.35 ± 352.49	560.54 ± 34.44	27.27	64.33	84.47	72.45	46.71
13	2-Furaldehyde	C5H4O2	Sweet, woody, almond, bready	9562.00	-	5.38 ± 0.01	-	-	-	-	<0.01	-	-	-
14	Benzaldehyde	C7H6O	Bitter almond	350.00	30.02 ± 0.66	135.05 ± 5.08	54.13 ± 3.37	47.02 ± 3.89	84.71 ± 2.88	0.09	0.39	0.15	0.13	0.24
15	(Z)-2-Pentenal	C5H8O	Fruity, sweet	1500.00	13.22 ± 1.19	252.70 ± 13.28	11.19 ± 0.26	10.60 ± 1.69	16.99 ± 2.99	0.01	0.17	0.01	0.01	0.01
16	(E)-2-Methyl-2-butenal	C5H8O	Fruity, Green	458.90	10.97 ± 1.78	13.54 ± 0.54	23.46 ± 1.58	17.91 ± 1.81	42.86 ± 4.09	0.02	0.03	0.05	0.04	0.09
17	(E)-2-Heptenal	C7H12O	Fatty, fruity	13.00	14.00 ± 2.52	19.72 ± 7.56	30.92 ± 6.83	38.16 ± 15.01	21.91 ± 1.49	1.08	1.52	2.38	2.94	1.69
18	1-Octanal	C8H16O	Green, citrus	0.70	42.90 ± 3.33	96.50 ± 6.17	67.79 ± 1.29	116.77 ± 8.71	136.38 ± 8.05	61.29	137.86	96.85	166.82	194.83
19	(E)-2-Hexenal	C6H10O	Green, fatty	17.00	7.19 ± 2.04	9.79 ± 0.37	18.27 ± 0.85	14.69 ± 0.45	18.71 ± 1.99	0.42	0.58	1.07	0.86	1.10
20	Heptaldehyde	C7H14O	Oily, green, citrus	3.00	62.57 ± 2.91	189.99 ± 13.6	245.66 ± 7.43	197.23 ± 21.78	242.94 ± 18.45	20.86	63.33	81.89	65.74	80.98
21	2-Methyl-2-pentenal	C6H10O	Aldehydes, soil, garlic, ripe cherries, fruity	290.00	21.96 ± 1.31	10.84 ± 0.89	13.06 ± 2.31	9.38 ± 1.40	17.42 ± 1.29	0.08	0.04	0.05	0.03	0.06
22	(E)-2-Pentenal	C5H8O	Green	1500.00	4.40 ± 1.51	6.16 ± 1.13	11.49 ± 1.33	5.98 ± 0.93	11.07 ± 1.44	<0.01	<0.01	0.01	<0.01	0.01
23	1-Nonanal	C9H18O	Fatty, green, citrus	1.00	36.70 ± 0.32	57.71 ± 2.51	50.84 ± 1.74	63.44 ± 3.83	82.98 ± 4.44	36.70	57.71	50.84	63.44	82.98
24	Propanal	C3H6O	Almond, cherry, green, fruity	37.00	27.55 ± 15.18	25.02 ± 3.60	37.52 ± 2.05	24.15 ± 15.67	31.98 ± 2.61	0.74	0.68	1.01	0.65	0.86
	Alcohols (11)													
25	Ethanol	C2H6O	Alcoholic	950,000.00	1177.13 ± 27.06	571.58 ± 12.68	910.10 ± 53.07	1168.34 ± 149.94	1251.94 ± 82.25	<0.01	<0.01	<0.01	<0.01	<0.01
26	3-Furanmethanol	C5H6O2	Special bitter and spicy	NA	103.64 ± 22.78	121.85 ± 11.27	155.60 ± 16.76	92.37 ± 20.44	186.35 ± 26.94	n.c.	n.c.	n.c.	n.c.	n.c.
27	Linalool	C10H18O	Citrus, rose, woody, blueberry	4.40	1984.89 ± 66.58	1011.88 ± 22.60	437.80 ± 11.12	935.00 ± 36.36	1298.76 ± 68.72	451.11	229.97	99.50	212.50	295.17
28	L-Menthol	C10H20O	Mint	2280.00	229.37 ± 14.01	375.18 ± 14.46	445.30 ± 20.29	475.37 ± 25.47	463.71 ± 26.31	0.10	0.16	0.20	0.21	0.20
29	1-Hexanol	C6H14O	Fresh, fruity, wine, sweet, green	5.60	7.55 ± 0.78	17.70 ± 0.26	41.06 ± 1.83	25.10 ± 1.63	56.49 ± 3.20	1.35	3.16	7.33	4.48	10.09
30	1-Pentanol	C5H12O	Balsamic	150.20	60.58 ± 7.02	74.55 ± 17.36	160.09 ± 25.11	107.33 ± 26.89	124.53 ± 6.85	0.40	0.50	1.07	0.71	0.83
31	1-Butanol, 3-methyl	C5H12O	Whiskey, banana, fruity	4.00	18.17 ± 0.99	23.05 ± 0.81	43.20 ± 4.41	36.27 ± 0.76	70.16 ± 4.70	4.54	5.76	10.80	9.07	17.54
32	1-Propanol	C3H8O	Alcoholic	8505.60	54.73 ± 17.31	43.05 ± 1.19	59.44 ± 1.73	446.08 ± 14.17	94.2 ± 15.96	0.01	0.01	0.01	0.05	0.01
33	2-Butanol	C4H10O	Fruity	330.00	11.82 ± 1.28	10.48 ± 0.30	21.70 ± 1.06	33.03 ± 0.53	33.67 ± 0.59	0.04	0.03	0.07	0.10	0.10
34	1-Propanol, 2-methyl	C4H10O	Bitter	7000.00	35.26 ± 5.10	19.55 ± 1.84	34.98 ± 3.36	94.66 ± 2.77	131.10 ± 6.78	0.01	<0.01	<0.01	0.01	0.02
35	Butanol	C4H10O	Alcohol-like, pungent	78	11.63 ± 2.84	10.93 ± 0.73	20.58 ± 0.93	19.63 ± 3.68	23.12 ± 1.76	0.15	0.14	0.26	0.25	0.30
	Esters (6)													
36	Acetic acid ethyl ester	C4H8O2	Fruity, sweet	5.00	127.03 ± 4.98	29.99 ± 2.78	48.69 ± 2.37	27.86 ± 1.07	654.43 ± 15.32	25.41	6.00	9.74	5.57	130.89
37	Acetic acid propyl ester	C5H10O2	Fruity	200.00	156.87 ± 58.68	191.51 ± 4.47	168.84 ± 6.85	121.99 ± 9.74	78.78 ± 13.93	0.78	0.96	0.84	0.61	0.39
38	2-Methyl-1-propyl acetate	C6H12O2	Fruity, raw pear and raspberrie	25.00	23.49 ± 5.42	12.95 ± 1.50	9.16 ± 0.60	11.91 ± 1.46	17.29 ± 1.28	0.94	0.52	0.37	0.48	0.69
39	Butanoic acid ethyl ester	C6H12O2	Pineapple, fruity, ester, whiskey	0.90	3.06 ± 0.97	4.72 ± 1.21	5.00 ± 0.58	10.44 ± 4.63	5.90 ± 0.32	3.40	5.24	5.56	11.60	6.56
40	Bornyl acetate	C12H20O2	Woody, pine, camphor	75.00	405.79 ± 14.93	233.03 ± 8.73	342.28 ± 16.79	392.17 ± 19.48	420.97 ± 26.41	5.41	3.11	4.56	5.23	5.61
41	Butanoic acid, 1-methylethyl ester	C7H14O2	Fruit, pungent	43.00	9.17 ± 0.79	3.97 ± 0.4	6.35 ± 0.18	5.37 ± 0.28	4.76 ± 1.16	0.21	0.09	0.15	0.12	0.11
	Ethers (6)													
42	Allyl disulfide	C6H10S2	Stinky, garlic	30.00	11.68 ± 3.73	74.36 ± 1.66	24.15 ± 4.16	15.07 ± 3.34	27.18 ± 2.4	0.39	2.48	0.80	0.50	0.91
43	Anethol	C10H12O	Anise, licorice, medicinal	15.00	7799.52 ± 221.77	5451.9 ± 273.86	7434.22 ± 118.15	6723.54 ± 320.01	7584 ± 319.65	519.97	363.46	495.61	448.24	505.60
44	Allyl sulfide	C6H10S	Garlic	100.00	6.07 ± 2.25	164.03 ± 17.35	7.05 ± 0.68	5.69 ± 1.26	9.28 ± 2.09	0.06	1.64	0.07	0.06	0.09
45	1,8-Cineol	C10H18O	Camphor, cool, mint	1.30	1542.28 ± 108.70	2177.89 ± 55.24	3188.79 ± 46.4	1984.49 ± 90	2610.45 ± 100.54	1186.37	1675.30	2452.92	1526.53	2008.04
46	1,4-Cineol	C10H18O	Cool, camphor, spice	1.10	197.31 ± 24.65	120.39 ± 17.46	129.71 ± 11.66	90.05 ± 12.46	157.24 ± 4.02	179.37	109.45	117.92	81.86	142.95
47	Dimethyl sulfide	C2H6S	Sulfury, oniony, sweet corn	0.30	81.33 ± 10.35	66.61 ± 2.64	120.12 ± 8.81	58.68 ± 12.22	115.62 ± 9.15	271.10	222.02	400.39	195.61	385.40
	Terpenes (11)													
48	α-Terpinolene	C10H16	Terpenic, green, woody	200.00	159.83 ± 29.59	125.84 ± 17.24	102.21 ± 14.43	96.47 ± 19.27	147.20 ± 13.41	0.80	0.63	0.51	0.48	0.74
49	β-Ocimene	C10H16	Herb, floral	34.00	53.84 ± 8.42	37.34 ± 5.77	41.98 ± 5.71	29.10 ± 10.44	57.01 ± 6.49	1.58	1.10	1.23	0.86	1.68
50	γ-Terpinene	C10H16	Pine, lemon	260.00	181.12 ± 42.5	116.36 ± 9.48	150.91 ± 17.06	101.47 ± 12.01	160.89 ± 19.72	0.70	0.45	0.58	0.39	0.62
51	Limonene	C10H16	Lemon, citrus, mint	10.00	102.79 ± 18.28	70.30 ± 9.81	48.23 ± 2.82	63.93 ± 15.29	94.39 ± 20.16	10.28	7.03	4.82	6.39	9.44
52	β-Pinene	C10H16	Woody, pine, minty, camphor	140.00	316.28 ± 99.74	281.09 ± 61.73	221.71 ± 44.29	201.78 ± 20.56	315.01 ± 53.74	2.26	2.01	1.58	1.44	2.25
53	β-thujene	C10H16	Woody, spicy, citrus	980.00	1368.30 ± 206.6	633.16 ± 109.2	777.68 ± 177.87	370.36 ± 70.01	907.46 ± 206.29	1.40	0.65	0.79	0.38	0.93
54	β-Myrcene	C10H16	Herbal, spice, mint	1.20	439.75 ± 115.13	446.59 ± 78.45	376.35 ± 70.5	312.18 ± 49.56	666.26 ± 106.39	366.46	372.16	313.62	260.15	555.22
55	α-Terpinene	C10H16	Lemony, citrusy	85.00	293.77 ± 84.76	181.35 ± 31.71	217.89 ± 54.73	135.23 ± 36.94	251.99 ± 44.96	3.46	2.13	2.56	1.59	2.96
56	δ-3-Carene	C10H16	Pungent, herb	770.00	67.25 ± 31.33	111.18 ± 25.73	47.48 ± 12.24	69.06 ± 13.41	137.92 ± 20.25	0.09	0.14	0.06	0.09	0.18
57	β-Phellandrene	C10H16	Turpentine, mint	8.00	225.97 ± 34.36	163.85 ± 18.64	137.36 ± 13.64	125.69 ± 19.16	197.58 ± 15.62	28.25	20.48	17.17	15.71	24.70
58	α-Pinene	C10H16	Camphor, pine, earthy	14.00	311.39 ± 109.21	140.50 ± 42.05	201.05 ± 65.85	160.00 ± 41.29	233.24 ± 55.98	22.24	10.04	14.36	11.43	16.66
	Others (2)													
59	2-Pentyl furan	C9H14O	Green, fat	6.00	31.11 ± 6.66	63.07 ± 17.61	65.73 ± 24.89	37.40 ± 14.17	105.20 ± 25.87	5.18	10.51	10.96	6.23	17.53
60	2,3,5,6-Tetramethylpyrazine	C8H12N2	Beef, fermented soy	2525.02	9.24 ± 0.47	-	45.78 ± 2.51	19.77 ± 2.53	51.73 ± 4.50	<0.01	-	0.02	0.01	0.02

Note: “^A^” the odor threshold of the compound is sourced from the website https://www.doc88.com/p-2039102934971.html (accessed on 2 September 2025). “NA” no data was reported. “-“ not detected; n.c., not calculated; no odor threshold available.

**Table 5:** Sensory evaluation of Beiwudu Hulatang from different brands.

	Appearance	Aroma	Mouthfeel	Taste	Overall Acceptability
Z	6.62 ± 0.98 ^c^	6.57 ± 1.17 ^b^	6.92 ± 1.18 ^b^	6.98 ± 1.32 ^b^	7.02 ± 1.05 ^b^
D	7.79 ± 0.73 ^ab^	8.39 ± 0.52 ^a^	7.27 ± 1.78 ^ab^	7.17 ± 1.08 ^ab^	7.91 ± 1.08 ^a^
W	7.94 ± 0.79 ^a^	8.26 ± 0.69 ^a^	8.19 ± 0.76 ^a^	8.12 ± 0.74 ^a^	8.48 ± 0.66 ^a^
G	7.24 ± 0.87 ^abc^	8.09 ± 0.64 ^a^	7.54 ± 1.01 ^ab^	7.58 ± 0.60 ^ab^	7.81 ± 1.12 ^ab^
S	6.97 ± 1.31 ^bc^	7.86 ± 1.05 ^a^	7.92 ± 0.86 ^ab^	7.26 ± 1.70 ^ab^	7.83 ± 1.26 ^ab^

Note: Different letters in the same column indicate a statistically significant difference (*p* < 0.05).

## Data Availability

The original contributions presented in this study are included in the article/Supplementary Materials. Further inquiries can be directed to the corresponding author.

## Supplementary Materials

The following supporting information can be downloaded at: https://www.mdpi.com/article/10.3390/foods14234054/s1, Figure S1: Process flow chart of sample Z. Table S1: Volatile components detected in Beiwudu Hulatang by GC-IMS.
