# Supplementary material for: Revealing the Flavor Characteristics of Beiwudu Hulatang Using Electronic Nose, Electronic Tongue Combined with GC-IMS and Sensory Analysis
Source: Foods. 2025 Nov 26;14(23):4054. doi: 10.3390/foods14234054 (PMC12692602; doi:10.3390/foods14234054)
Supplement: Supplementary file 1 [file foods-14-04054-s001.zip › foods-3985011-supplementary.pdf]

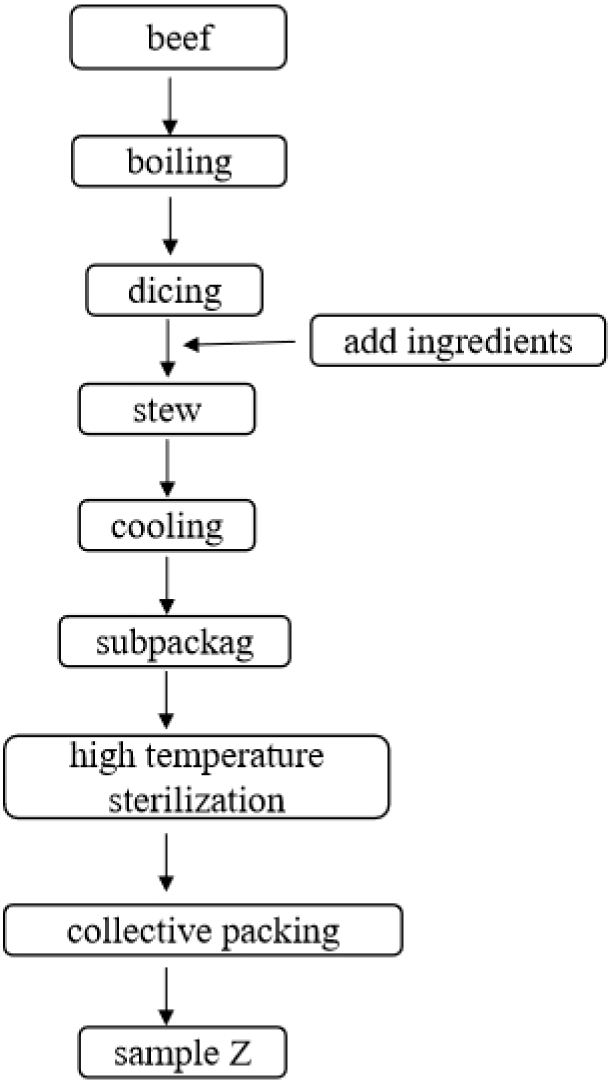

Figure S1. Process flow chart of sample Z.

Table S1. Volatile components detected in Beiwudu Hulatang by GC-IMS.

Count	Compound	CAS#	Formula	MW	RI	Rt [sec]	Dt [a.u.]
1	Acetic acid	C64197	C2H4O2	60.1	1473.8	907.788	1.05094
2	1-Hydroxy-2-propanone	C116096	C3H6O2	74.1	1314.5	655.259	1.09898
3	3-Methyl butanal	C590863	C5H10O	86.1	922.3	207.123	1.39734
4	2-Propanone	C67641	C3H6O	58.1	818.6	168.491	1.11031
5	2-Butanone	C78933	C4H8O	72.1	905.9	199.861	1.2388
6	Ethanol	C64175	C2H6O	46.1	938.7	216.128	1.13414
7	2-Furaldehyde	C98011	C5H4O2	96.1	1469.3	899.438	1.08575
8	Propanoic acid	C79094	C3H6O2	74.1	1563.1	1089.641	1.11369
9	Benzaldehyde	C100527	C7H6O	106.1	1509.7	976.866	1.15123
10	2-Butanone, 3-hydroxy	C513860	C4H8O2	88.1	1296.6	631.705	1.09353
11	(Z)-2-Pentenal	C1576869	C5H8O	84.1	1119.8	364.691	1.08986
12	(E)-2-Methyl-2-butenal	C497030	C5H8O	84.1	1100.8	342.069	1.09407
13	3-Furanmethanol	C4412913	C5H6O2	98.1	1661.5	1332.714	1.09804
14	(E)-2-Pentenal	C1576870	C5H8O	84.1	1135.8	384.81	1.10995
15	Isomenthone	C491076	C10H18O	154.3	1486.9	932.403	1.35087
16	Allyl disulfide	C2179579	C6H10S2	146.3	1473.1	906.348	1.19714
17	Linalool-M	C78706	C10H18O	154.3	1566.9	1098.265	1.22122
18	Linalool-D	C78706	C10H18O	154.3	1567.5	1099.59	1.7032
19	L-Menthol	C2216515	C10H20O	156.3	1683.3	1393.548	1.23469
20	Anethol-M	C104461	C10H12O	148.2	1960.8	2458.381	1.21354
21	Anethol-D	C104461	C10H12O	148.2	1959.4	2451.79	1.74102
22	1-Hexanol	C111273	C6H14O	102.2	1370.8	735.172	1.32896
23	2-Methyl-2-hepten-6-one	C110930	C8H14O	126.2	1351.6	706.975	1.17288
24	(E)-2-Heptenal	C18829555	C7H12O	112.2	1336.5	685.355	1.25568
25	1-Octanal-M	C124130	C8H16O	128.2	1300.5	636.8	1.43629
26	1-Octanal-D	C124130	C8H16O	128.2	1302.3	639.134	1.81558
27	2-Octanone	C111137	C8H16O	128.2	1294.8	629.33	1.33395
28	α-Terpinolene	C586629	C10H16	136.2	1288.2	617.658	1.21203
29	1-Pentanol-M	C71410	C5H12O	88.1	1261.4	570.037	1.25417
30	1-Pentanol-D	C71410	C5H12O	88.1	1261.9	570.97	1.52058
31	β-Ocimene	C13877913	C10H16	136.2	1258.9	565.872	1.20687
32	γ-Terpinene	C99854	C10H16	136.2	1246.2	544.631	1.21417
33	2-Pentyl furan	C3777693	C9H14O	138.2	1237.1	530.014	1.24685
34	(E)-2-Hexenal	C6728263	C6H10O	98.1	1224.4	510.221	1.18673
35	1-Butanol, 3-methyl	C123513	C5H12O	88.1	1212.4	492.254	1.24685
36	Limonene	C138863	C10H16	136.2	1204	480.056	1.21165
37	Heptaldehyde-M	C111717	C7H14O	114.2	1186.6	455.627	1.37134
38	Heptaldehyde-D	C111717	C7H14O	114.2	1186.3	455.274	1.69129
39	2-Heptanone	C110430	C7H14O	114.2	1183.5	451.412	1.26641
40	Acetic acid ethyl ester	C141786	C4H8O2	88.1	886.1	192.266	1.33286
41	Pentanal-M	C110623	C5H10O	86.1	986.2	244.595	1.22358
42	Pentanal-D	C110623	C5H10O	86.1	983.9	243.141	1.416
43	Acetic acid propyl ester-M	C109604	C5H10O2	102.1	977.6	239.182	1.16625
44	Acetic acid propyl ester-D	C109604	C5H10O2	102.1	977.6	239.182	1.47528
45	2-Methyl-1-propyl acetate	C110190	C6H12O2	116.2	1013.5	264.445	1.23522

46	1-Propanol-M	C71238	C3H8O	60.1	1040.1	285.497	1.1125
47	1-Propanol-D	C71238	C3H8O	60.1	1039.8	285.196	1.2612
48	2-Butanol	C78922	C4H10O	74.1	1025.5	273.768	1.1555
49	Butanoic acid ethyl ester	C105544	C6H12O2	116.2	1062.1	304.1	1.21006
50	Butanoic acid, 1-methylethyl ester	C638119	C7H14O2	130.2	1070.9	311.9	1.25764
51	1-Propanol, 2-methyl	C78831	C4H10O	74.1	1098.3	339.269	1.1749
52	β-Pinene-M	C127913	C10H16	136.2	1097	337.731	1.20958
53	β-Pinene-D	C127913	C10H16	136.2	1097.7	338.585	1.67781
54	β-Thujene-M	C28634891	C10H16	136.2	1112.1	355.336	1.21759
55	β-Thujene-D	C28634891	C10H16	136.2	1113.8	357.387	1.63645
56	Allyl sulfide	C592881	C6H10S	114.2	1149.5	402.939	1.11914
57	Butanol	C71363	C4H10O	74.1	1146.6	399.127	1.18995
58	2-Methyl-2-pentenal	C623369	C6H10O	98.1	1160.5	418.142	1.15951
59	β-Myrcene-M	C123353	C10H16	136.2	1158.4	415.308	1.2147
60	β-Myrcene-D	C123353	C10H16	136.2	1157.7	414.27	1.6848
61	α-Terpinene-M	C99865	C10H16	136.2	1173	436.21	1.21198
62	α-Terpinene-D	C99865	C10H16	136.2	1171.9	434.592	1.70786
63	δ-3-carene	C13466789	C10H16	136.2	1141.4	392.126	1.21198
64	β-Phellandrene-M	C555102	C10H16	136.2	1192.9	464.393	1.21171
65	β-Phellandrene-D	C555102	C10H16	136.2	1192	463.138	1.64843
66	1,8-Cineol-M	C470826	C10H18O	154.3	1202	477.211	1.31366
67	1,8-Cineol-D	C470826	C10H18O	154.3	1202.4	477.733	1.71462
68	1,4-Cineol	C470677	C10H18O	154.3	1193	464.551	1.31073
69	2,3,5,6-Tetramethylpyrazine	C1124114	C8H12N2	136.2	1466.1	893.499	1.21343
70	α-Pinene-M	C80568	C10H16	136.2	1023.5	272.195	1.2126
71	α-Pinene-D	C80568	C10H16	136.2	1025.5	273.724	1.66192
72	Dimethyl sulfide	C75183	C2H6S	62.1	763.3	151.201	0.95136
73	1-Nonanal	C124196	C9H18O	142.2	1398.7	778.509	1.50433
74	Propanal	C123386	C3H6O	58.1	785.1	157.776	1.13997
75	Bornyl acetate	C76493	C12H20O2	196.3	1599.4	1173.759	1.21707
76	1	unidentified	*	0	1398.2	777.57	1.50401
77	2	unidentified	*	0	1131.1	378.841	1.26113
78	3	unidentified	*	0	1013.9	264.745	1.28896
79	4	unidentified	*	0	1561.3	1085.671	1.43035
80	5	unidentified	*	0	1389.2	763.37	1.3237
81	6	unidentified	*	0	1344.3	696.401	1.4447
82	7	unidentified	*	0	1257	562.597	1.12138
83	8	unidentified	*	0	1074.1	314.759	1.39357
84	9	unidentified	*	0	1137.7	387.364	1.12684
85	10	unidentified	*	0	1146.9	399.427	1.57197
86	11	unidentified	*	0	1240.4	535.352	1.29865

Note: "CAS#" an alias of CAS number which indicates the unique numerical identification number of a substance. "MW" the molecule weight of the volatiles. "RI" retention Index. "Rt" retention time. "Dt" drift time in the drift tube.
